# Inflammatory Mediators Involved in Conjunctival and Corneal Remodeling of Vernal Keratoconjunctivitis

**DOI:** 10.3390/life16071087

**Published:** 2026-06-29

**Authors:** Nicholas J. Karbach, Fiza Tariq

**Affiliations:** Pennsylvania College of Optometry, Drexel University, Elkins Park, PA 19027, USA; njk66@drexel.edu

**Keywords:** eosinophils, mast cells, IL-4, IL-5, IL-13, keratoconus, MMP-9, Th2 cells, vernal keratoconjunctivitis, anti-inflammatory medicines, corneal remodeling

## Abstract

Vernal keratoconjunctivitis (VKC) is a chronic condition that causes remodeling of the cornea and conjunctiva through recurring episodes of allergic inflammation of the ocular surface. This can lead to corneal scarring, keratoconus, and chronic conjunctival papillae. However, the details of the immunopathological processes behind these remodeling changes are not completely understood. Despite involving IgE-modulated mechanisms, about half of patients with VKC test negative on systemic allergy tests. This calls for the need to understand the common and novel inflammatory mediators involved in the pathogenesis of VKC development and severity levels. Eye rubbing stimulates the release of inflammatory cytokines TNF-alpha, IL-4, IL-5, and IL-13 and remodeling enzymes MMP-1, MMP-3, MMP-9, and MMP-10, which drive a dysregulated cycle of stromal tissue remodeling that leads to progressive ectasia. Eosinophilic activity is driven by CCL11 and ICAM-1 and eye rubbing, which leads to degranulation and the release of EMBP, ECP, and MMP-9. These inflammatory mediators drive the remodeling changes that lead to corneal scarring and ectasia. The purpose of this comprehensive review paper is to shed light on common and novel immunological mediators that help us further understand VKC and eventually lead to the discovery of more effective and targeted treatment options.

## 1. Introduction

VKC is a severe chronic allergic condition that causes permanent ocular surface remodeling changes through recurring episodes of allergic inflammation [1,2]. VKC typically occurs in males in the first and second decade of life and can occur year-round, especially in warm climates [2,3,4]. The disease itself is self-limiting; however, chronic and recurring allergic episodes of papillary conjunctivitis, chemosis, redness, and severe itching can lead to the formation of giant papillae, chronic conjunctival injection, corneal epithelial erosions, corneal vascularization, limbal hypertrophy, shield ulcers, corneal scarring, and keratoconus [5]. Conjunctival papillae on the superior corneal limbus are a hallmark sign of VKC and are referred to as Horner–Trantas dots [4]. These lesions are aggregations of eosinophils and dead epithelial cells [3]. These sequelae can eventually lead to vision loss through corneal scarring [6,7]. In children, VKC has the potential to impair future quality of life [5,8,9]. The chronic remodeling changes of this disease are the main driver of functional loss and quality of life loss. These sequelae occur due to a variety of immunopathological interactions, which, when summarized, provide a clearer picture of what pharmacotherapeutic targets can be prioritized in future research.

The pathophysiology of VKC is driven by the type 2 helper T lymphocyte (Th2) immune response and involves both the innate and acquired immune systems [10,11]. The inflammatory response to environmental antigens is mediated by IgE, IL-4, IL-5, IL-13, TNF-α, mast cells, eosinophils, and Th2 lymphocytes [12].

VKC commonly presents in young children and self-resolves around puberty; however, according to a few case reports and retrospective analyses, some cases can persist into adulthood or develop after puberty [13]. It is also more commonly found in male subjects compared to female subjects [14]. VKC may be present alone or in conjunction with atopic conditions such as allergic dermatitis, asthma, and allergic rhinitis [15]. Allergic conjunctivitis has been estimated to impact 6–30 percent of the general population, and in up to 30 percent of children, there is some level of association with allergic rhinitis [16]. In patients with ocular allergies 9% are affected by VKC, increasing to 22% in patients under age 18 [17].

There is significant variance in VKC among ethnic groups and in different parts of the world. High prevalence rates are especially noted in warm and dry climates such as the Middle East, central and southern Africa, and Mediterranean countries [18]. Ophthalmological-based examinations have been performed in many African countries to estimate VKC prevalence. In 2007 in Rwanda, the prevalence of VKC was 4% in school-aged children between the ages of 7 and 14 years of age [19]. In 2014 in Nigeria, a high prevalence of 18.1% of VKC was reported in school-aged children between the ages of 4 and 15 years of age [18]. In another 2015 survey in Ethiopia, the prevalence of VKC was 5.8% for children under the age of 18 [18]. The most recent survey in 2018 indicated an increased prevalence to 11.1% among children in Ethiopia [14].

A questionnaire-based survey was completed by ophthalmologists in six European countries to estimate the prevalence of VKC in Western Europe [20]. The survey findings indicated that VKC prevalence was 3.2/10,000 inhabitants [20]. They also found the prevalence of corneal complications secondary to VKC to be 0.8/10,000 inhabitants [20]. There is agreement on these low VKC prevalence levels in European countries.

The prevalence of VKC varies greatly depending on the study. Using patient-based surveys, the ratio of VKC patients amongst all ocular allergy patients was 10.8% in Thailand in 2000 [21]. In Japan, the ratio of VKC was 3.8% in ocular allergy patients in 2003 [18]. Moreover, in Italy, a cross-sectional study using an in-office questionnaire concluded a prevalence of 9% of VKC, with a higher prevalence in patients under the age of 18 [17]. It is important to note that these large variations in ratio may not reflect actual prevalence differences because they are from various regions and years, and because of differences due to sampling bias, the medical resources utilized, and cultural differences [18]. 

In a study on Rwandan school children, those with VKC were 5x more likely to not attend school in a 3-month period than those without VKC [19]. Pediatric quality of life surveys showed lower scores for children with allergic conjunctivitis with VKC scoring lower than all other subtypes [22]. Nearly 5% of VKC patients have been found to exhibit steroid-related ocular hypertension or glaucoma with nearly 25% requiring surgery [23].

This narrative review aims to present the inflammatory markers behind the conjunctival and corneal remodeling found in VKC patients. Its purpose is to identify Ig-E and non-IgE-mediated immune mechanisms that underly VKC and provide a detailed analysis of each inflammatory mediator that is involved in VKC pathogenesis. It is crucial to understand these biological key players so we can utilize them to form novel treatment options and guide clinical management of the disease [24].

## 2. Methods of Research

A literature review was performed through the PubMed and ScienceDirect Journals electronic databases on 15 November 2025. Both PubMed and ScienceDirect are excellent sources of peer-reviewed scientific research. The key terms “vernal keratoconjunctivitis” and different combinations with the terms “conjunctiva”, “cornea”, “pathophysiology”, “immunology”, “eosinophils”, and “mast cells” were used. All abstracts from studies published after 1 January 2000 were reviewed and appropriately included. All articles not written in English were excluded. Only peer-reviewed articles, including systematic reviews were included. There were no limitations based on study region or age/gender. The reference lists of the selected papers were also manually screened to identify any articles not found directly in the electronic databases. The different searches provided 20 relevant papers, which formed the foundation of this paper. The bibliography was expanded as necessary to comprehensively substantiate narrative connections.

## 3. Discussion

### 3.1. Overview of Immune and Pathological Mechanisms Underlying Immune Activation in VKC

An allergic reaction to environmental allergens begins with the uptake of antigens by tissue-resident dendritic cells that present to naïve CD4+ T cells, which proliferate and differentiate into helper T cells. This begins a cascade of events, including the activation, clonal expansion, and differentiation of allergen-specific B cells into IgE-producing plasma cells. The released ImmunoglobulinE (IgE) then binds to receptors on conjunctival mast cells, which prime the mast cells. Later, re-exposure of those primed mast cells to allergens leads to degranulation to release histamine and other mediators [25].

Th2 lymphocytes and eosinophils are recruited to the ocular surface in response to chemokines released by degranulated mast cells. The ocular surface epithelial cells express intracellular adhesion molecules-1 (ICAM-1). The interaction between the lymphocyte function-associated antigen-1 (LFA-1) which is expressed on these immune cells, and (ICAM-1) which is expressed on the epithelial cells, enhances the recruitment of the Th2 lymphocytes and eosinophils [26]. Furthermore, fibroblasts express ICAM-1 and produce C-C motif chemokine ligand-17 (CCL17) to also mediate the recruitment of Th2 cells. (CCL11), released also by fibroblasts, mediates the recruitment of eosinophils [25]. This production of ICAM-1 is stimulated by TNF-α and IL-4 [26,27]. Th2 cells produce IL-4, IL-5, IL-9, and IL-13. IL-5 activates and recruits eosinophils, and IL-9 recruits more mast cells [3,10,28]. Eosinophils are also recruited to the site of inflammation through the upregulation of ICAM-1 and eotaxin-1 in conjunctival fibroblasts and epithelial cells [7,12,26,28].

**Figure 1:**
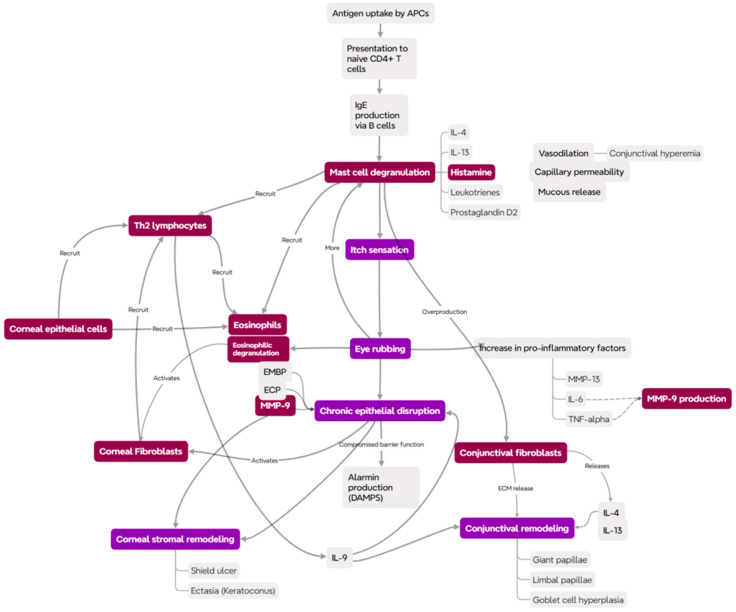
Mechanistic model for corneal and conjunctival remodeling in VKC—highlighting key inflammatory mediators. This figure demonstrates the key importance of the axis of itch sensation, eye rubbing, and chronic epithelial disruption as a core mechanical instigator of the feedback loop that leads to chronic disease. The cascading effects of mast cell and eosinophilic degranulation are shown. The importance of MMP-9 and IL-9 in the propagation of tissue remodeling is also illustrated [35,36,37,38,39,40,41,42].

Degranulating mast cells release cytokines IL-4, IL-13, and histamine, which causes the pathognomonic itch sensation on the ocular surface [26,29]. Mast cells also release leukotrienes, which induce vasodilation and capillary permeability [26], and prostaglandin D2 (PGD2), which induces vasodilation, ocular itch sensation, and mucous release by goblet cells [26]. Platelet-activating factor (PAF) and IL-5 from mast cells induce further chemotaxis of eosinophils [26,28]. Eye rubbing in response to itching causes further degranulation of mast cells and disrupts the corneal epithelium and bowman’s layers, which furthers the inflammatory cascade (see Figure 1) [30].

Eosinophils are largely responsible for the tissue damage and remodeling in VKC [31]. Eosinophil degranulation releases eosinophil cationic protein (ECP), eosinophilic major basic protein (EMBP), eosinophil peroxidase, and matrix metalloproteinases (MMP) [12,26]. These cytotoxic chemicals disrupt the corneal epithelium, inhibiting healing and promoting activation of fibroblasts in the corneal stroma [7,26,32]. These compounding effects of eye rubbing, corneal mechanical damage and eosinophil degranulation, create a dysregulated cycle of destruction and remodeling that can lead to corneal scarring and ectasia.

Corneal epithelial damage compromises the barrier function of the cornea and helps induce the type 2 immune response through the production of alarmins. Alarmins, also known as damage-associated molecular patterns (DAMPs), are a broad category of epithelial-derived signal molecules that trigger allergic inflammation [7,33]. This group includes IL-1α, IL-25, IL-33, and thymic stromal lymphopoietin (TSLP), defensin, and other substances [7,34]. In the setting of frequent eye rubbing, DAMPs/alarmins exacerbate the inflammatory cascade, interfering with homeostasis in VKC and contributing to the corneal and conjunctival changes seen in VKC [7]. Thus, eye rubbing functions both as a trigger for inflammation as well as an exacerbator, furthering the cycle of inflammation (see Figure 1).

### 3.2. Mechanisms Underlying VKC Ocular Signs and Symptoms

Patients with VKC often present with intense symptoms of ocular itching, which occurs due to the interaction of histamine, primarily released by mast cells, and the histamine 1 (H1) receptors on the conjunctival sensory nerve fibers [35,36]. Damaged epithelial cells, mast cells, eosinophils, and Th2 cells release the cytokine IL-31, which, in addition to IL-4 and IL-13, is also responsible for intense itching sensation [37]. Another common clinical finding in VKC is conjunctival hyperemia and perilimbal chemosis, which occurs due to the interaction of the mast cells and released histamine interacting with the H1 receptors on conjunctival blood vessels [38]. This histamine-to-H1-receptor interaction on the blood vessels leads to vasodilation and capillary leakage, which present hyperemia and chemosis, respectively [38]. Leukotrienes have a similar effect to histamines on the blood vessels and contribute to the conjunctival hyperemia and chemosis. Moreover, the prostaglandins E2 and D2 (PGE2 and PGD2) are involved in further increasing vascular leakage and vasodilation on the conjunctival blood vessels, which further intensifies the ocular itching sensation [36,39]. IL-4 also plays a role in vasodilation and vascular permeability by interacting with its receptors on the conjunctival vascular endothelial cells, which upregulates vascular cell adhesion molecule 1 (VCAM-1), furthering the signs of conjunctival hyperemia and chemosis [40]. Mucus discharge can also be found in VKC patients, and this is primarily due to the involvement of IL-13 and histamine [36]. IL-13 release can lead to the hyperplasia of goblet cells on the conjunctival epithelium, as well as cause hypersecretion of mucin from these activated goblet cells [41]. Other players include IL-9 and histamine, which can cause hyperplasia of goblet cells, and these goblet cells can cause the release of mucin, respectively [42].

One of the hallmark and severe ocular signs of VKC is cobblestone-like giant papillae. This papillary hyperplasia most commonly affects the upper tarsal conjunctiva and the limbal bulbar conjunctiva [43]. Conjunctival fibroblasts are responsible for releasing histamine, IL-4, and IL-13, which causes overproduction of more fibroblasts, and the release of extracellular matrix from these fibroblasts [26,44]. Th2 cells, Th9 cells, eosinophils, and mast cells can release IL-9, which combines with IL-4 to cause remodeling of the conjunctival tissue [45]. Thus, activated conjunctival fibroblasts play a key role in fibroproliferative changes and tissue remodeling of the conjunctiva, which are the giant papillae clinically observed [26,46]. Histopathological assessment of these papillae reveals a hyperplastic epithelium with a fibrovascular core and a variety of inflammatory cells infiltrated into the subepithelial layer of the conjunctival tissue [46]. The hyperproliferation and release of extracellular matrix from activated fibroblasts, as well as the influx of inflammatory cells and hypervascularization, are responsible for the presence of cobblestone-shaped giant papillae [5,46]. Moreover, papillae in the limbal region of the bulbar conjunctiva tend to have a confluent, gelatinous appearance [47].

In severe and chronic cases of VKC, corneal involvement is typically found, which, if left untreated, can lead to permanent vision loss. Corneal findings in VKC patients include punctate corneal keratitis, neovascularization, microbial keratitis, shield ulcers, stromal scarring, limbal stem cell deficiency, and keratoconus [48,49]. The pathogenesis of corneal degradation is due to the toxic mediators released due to the degranulation of eosinophils, including ECP, EBMP, and MMP-9 [50]. These mediators cause degradation of the corneal basement membrane, which leads to persistent epithelial keratopathy—a pathognomonic sign in severe cases of VKC [51]. IL-9 can also interact with corneal epithelial cells and cause a breach in the epithelium barrier function, which leads to the development of punctate epithelial erosions on the corneal surface [52]. As a result of persistent corneal epithelial compromise that extends deeper into Bowman’s layer and breaks down collagen in the corneal stroma, another pathognomonic sign of VKC can form a shield ulcer [53]. Shield ulcers are oval-shaped corneal epithelial defects with elevated margins and are a vision-threatening complication of VKC that can cause irreversible damage if left untreated [53].

Keratoconus is a chronic progressive corneal ectasia that occurs in up to 26.7% of patients with VKC [12]. The association between keratoconus and VKC may be due to the shared association with eye rubbing or a combination of shared mechanical, genetic, and immunopathological factors [54]. For instance, vigorous eye rubbing has been shown to increase the level of pro-inflammatory factors such as IL-6 and MMP-13, which increase production of MMP-9, leading to pathological stromal remodeling and ectasia [55]. A clear pathophysiological model of keratoconus has remained elusive despite numerous factors such as atopy and eye rubbing being well-established [56].

Additionally, excessive eosinophil infiltration and dead epithelial cells can accumulate on the limbal cornea as these opaque, yellow-white deposits and lead to another pathognomonic sign in VKC called Horner–Trantas dots [57]. Other signs in VKC include white lipid deposits in the peripheral cornea, which present due to longstanding vascular permeability in the limbal bulbar conjunctiva and are known as pseudogerontoxon [48,57].

### 3.3. Key Markers of Chronic Corneal Remodeling in VKC

As alluded in the previous section, corneal remodeling is a hallmark characteristic of VKC and can be detrimental to a patient’s vision if left untreated. Histological studies of VKC patients with advanced limbal hypertrophy have shown epithelial hyperplasia, an absent Bowmans layer, and thickened and occasionally vascularized stroma with chronic inflammatory cells, infiltrates, or eosinophils [6]. Biomechanical studies have shown that VKC patients have corneas that are thinner and more flexible [58]. Wang et al. studied corneal morphology and biomechanical indices, including corneal irregularity and asymmetry and a thinner corneal epithelium, which are all correlated with eye rubbing frequency and signs of ocular allergy [59]. In patients that go on to develop keratoconus, epithelial migration forms an abnormal topography where the epithelial layer is thin at the cone and thickens in a donut shape around the cone. This is caused by dysregulation of cell proliferation and migration, where migration to the middle cornea is increased and migration from the middle topographic region to the central region is decreased [60]. Keratoconus is increasingly being understood as a dysregulation of homeostatic stromal repair mechanisms mediated by inflammatory cytokines [61,62,63].

MMPs are a group of enzymes that play a significant role in tissue repair by breaking down injured tissue degradation and apoptosis to allow for remodeling [64,65,66]. They are restricted by endogenous inhibitors known as tissue inhibitors of metalloproteinase (TIMP) [67,68]. MMPs are produced by corneal epithelial cells, fibroblasts, and eosinophils in response to corneal epithelial damage [68]. VKC causes upregulation of multiple MMPs, and downregulation of TIMP, and dysregulation of lysyl oxidase (LOX)—an enzyme that facilitates collagen cross-linking [69]. These tear proteome abnormalities overlap with keratoconus and represent shared dysregulated tissue remodeling pathways. Tear samples in VKC show elevated levels of MMP-1, MMP-2, MMP-3, MMP-8, MMP-9, and MMP-10 [10]. KC tears have elevated levels of MMP-1 [64], MMP-2 [64], MMP-3 [64], MMP-7 [64,70], MMP-9 [30,64,70], MMP-13 [30,64,70], and IL-6 [71,72] and reduced levels of IL-10 and TIMP-1 [30].

MMP-9 is a gelatinase the degrades type IV collagen which is found in the corneal epithelial basement membrane and Descemet’s layer [55,73,74]. MMP-9 is elevated in both VKC and KC and has been found to inhibit corneal re-epithelialization, exacerbate extracellular matrix degradation, and precipitate sterile corneal ulceration [26,64,71]. It is produced by the corneal epithelial cells, fibroblasts, and eosinophils through IL-1b, IL-6, and TNF-α [12,55,64]. Release of MMP-9 (as well as MMP-1, MMP-3, and MMP-10) is triggered by insult to the corneal epithelium [64,68]. Desiccating stress leads to increased MMP production as well as increased corneal epithelial permeability. This breach in the epithelial barrier function allows remodeling enzymes in the tear film to reach the stroma and create remodeling changes there as well [68].

MMP-9 regulation is of interest in the treatment of VKC. MMP activity can be regulated in three places: transcription, pro-enzyme activation, and TIMP activity [68]. Chymase cleaves pro-MMP-9 into MMP-9 and is directly implicated in increased levels of MMP-9 in VKC and may serve as a therapeutic target [75]. TIMP-1 inhibits MMP-9 [55,76,77]. MMP-2 is inhibited by TIMP-2 [77]. Cyclosporine-A can be used to inhibit MMP-9 as well as IL-6 and TNF-α in both VKC and KC [64,72].

Lysyl oxidase (LOX) is an enzyme that catalyzes the formation of precursors for collagen cross-linking, a process that is dysregulated in both VKC and KC [30,69,71,78]. Cross-linking of stromal collagen is a biomechanical protector against corneal ectasia [30]. Confocal microscopy reveals decreased cross-linking patterns in allergic conjunctivitis [69]. Gene expression of LOX is decreased in correlation with an increase in TNF-α, an increase in IL-6, and a decrease in IL-10, particularly [30,71] in regions where Bowman’s membrane is broken or absent, which can occur from chronic eye rubbing [30,60,71,78].

### 3.4. New Insights: Role of Innate Immunity in VKC

The ocular surface structures, including the eyelids, lacrimal gland, tear film, cornea, and conjunctival epithelia, play a key role in providing a first line of defense against foreign pathogens, toxic stimuli, and allergens [79]. All these structures act as a non-specific surveillance system, activating the adaptive immune response through the release of various cell mediators. The orbit and the eyelid protect the eye against trauma, and the tears, cornea, and conjunctiva glycocalyx provide additional barriers [80,81]. There is increasing evidence that the conjunctival mucosal epithelia play an active role in the ocular surface inflammatory response and in initiating the innate immune system [77]. Tears, produced by the lacrimal gland, can flush out particles from the ocular surface and protect against foreign material through antimicrobial proteins such as lactoferrin, lysozyme, lipocalin, β-lysin, and specific secretary immunoglobins sIgA and sIgG [78].

All of these ocular surface structures and their cells and mechanisms protect the ocular surface for the first few minutes to hours and form an immediate response, initiating a cascade of defense mechanisms involving the recruitment of both resident and immune cells [82]. In response to inflammatory mediators, the ocular surface epithelia release a mix of chemokines, cytokines, prostaglandins, leukotrienes, and growth factors [79,81]. In comparison to the normal conjunctiva, VKC specimens showed an increase in intercellular adhesion molecule-1 (ICAM-1), intercellular adhesion molecule-3 (ICAM-3), lymphocyte function-associated antigen-1 (LFA-1), and very late activation antigen-4 (VLA-4) [83]. Another study that aimed to reduce the signs and symptoms of VKC using probiotic eye drops found that after 4 weeks of treatment, there was decreased expression of ICAM-1 and toll-like receptor 4 (TLR4) on the ocular surface of treated patients [84]. This study highlights another key factor that mounts the initial, immediate immune response against pathogens, i.e., through pathogen recognition using specific receptors or, in other words, toll-like receptors (TLRs).

The ocular surface epithelia play an important role in mounting the immune response in VKC through the expression of membrane receptors called TLRs [85]. TLRs are pattern recognition receptors that recognize pattern-associated molecular patterns (PAMPs) on bacteria, fungi, viruses, and protozoa and stimulate appropriate adaptive immune responses [72]. For their unique role in recognizing patterns from commensal bacteria, their role in ocular surface infectious disease has been thoroughly examined and recognized [86]. However, there is also emerging evidence of the TLRs’ key role in ocular allergic diseases, highlighting the role they play in the interconnectedness between innate and adaptive immune response [87]. Several studies have linked TLRs to play an important role in ocular surface allergic disease, such as VKC, and new findings continue to support their presence in the innate immune response [88,89].

TLRs may be expressed by a variety of cells on the ocular surface. TLRs may be classified based on their localization (intracellular or surface receptor) or by their respective ligands, which are structures presented by foreign pathogens (not expressed by the host), allowing the recognition of non-self vs. self in the innate immune response [90]. TLR activation leads to the release of cytokines, recruitment of immune cells, and even modulation of corneal healing in response to environmental allergens and pathogens [91]. Table 1 summarizes the common ligands recognized by TLRs found to be active in VKC patients and the pathogens that allow for their expression [82,89].

A recent 2022 study found decreased conjunctival epithelium expression of TLR4 and TLR9 in children with VKC compared to healthy subjects, suggesting that TLRs may contribute to the inhibition of the allergic reaction [91]. TLR9 has also been previously found to be downregulated in the conjunctival epithelium of VKC patients, further supporting its protective role in allergic immune response in VKC [88,92]. Conversely, other studies have found TLR4 to be upregulated in the presence of infiltrating immune cells in the conjunctiva of VKC patients [93]. Another study found that the enhanced expression of TLR4 led to the overproduction of pro-inflammatory cytokines and chemokines that activate and recruit T cells and further exacerbate the immune response [94]. Another study found the expression of TLR4 on the basal and wing cells but not the superficial epithelial cells of the human corneal tissue sections [95]. This distinct expression of the TLR4 in deeper layers of the cornea suggests that TLR4 may be only activated when there is a breach in the epithelium and thus prevents unnecessary inflammatory responses [95,96]. Other TLRs with overexpression in VKC patients with active inflammation were TLR2 and TLR8 as well [97]. Stimulation of TLR2 and TLR4 on corneal fibroblasts by Pam3CSK4 or lipopolysaccharide (LPS) challenge caused a significant increase in IL-6 and TNF-alpha secretion from macrophages., indicating the linking role that TLRs have in activating corneal fibroblasts [97].

Another cell that has shown to play a role in VKC pathogenesis is the human natural killer (NK) cell, also known as the innate immune effector cell [98]. NK cells can lyse tumorigenic and virally infected cells without prior sensitization to the antigen and may be induced to release Th2 cytokines and chemokines, including IL-5, IL-8, IL-13, CCL3, CCL4, and CCL5 [99]. When compared with healthy subjects, there was a significant increase in the conjunctiva of NK cells in VKC patients [100]. Thus, in addition to lymphocytes, mast cells, and eosinophils, NK cells can impact the pathogenesis and play a significant role in infiltrating the conjunctiva in VKC patients [98,100]. NK cells exert a regulatory effect on macrophages and dendritic cells by engaging in cross-talk through TLR-9 interactions [101]. Additionally, blocking of NK cell function in wounded corneas resulted in a 114% higher rate of neutrophil infiltration. NK cells have also been shown to enhance MMP-1-induced collagen degradation as they infiltrate tissues via CXCL12-aided migration [102].

By increasing a deeper understanding of the innate immune response system in VKC, including the TLRs and NK cells, novel therapies can be developed that provide timely relief and avoid long-term complications.

## 4. Conclusions

VKC causes corneal and conjunctival remodeling that can lead to chronic giant conjunctival papillae, cornea scarring, and keratoconus. The release of inflammatory cytokines TNF-alpha, IL-4, IL-5, and IL-13 and remodeling enzymes MMP-1, MMP-3, MMP-9, and MMP-10 plays a major role in the pathology of this condition as well as sequelae such as shield ulcer and keratoconus. The role of innate immunity and the ocular surface biome in VKC development is an emerging area of understanding and could represent additional treatment vectors. The role of TLRs in response to allergens may be another area of focus for a better understanding of VKC. Modulation of TLR expression could be used to treat the dysregulated immune response seen in VKC. Eosinophilic activity in conjunction with eye rubbing is driven by CCL11 and ICAM-1, and eye rubbing leads to degranulation and the release of EMBP, ECP, and MMP-9 [72]. Since effective preventative treatments for both VKC and its sequelae are lacking, further investigation is warranted to understand the inflammatory and immunological mechanisms that lead to the chronic failure of homeostasis to hone in on more effective treatments. If the recurrent cycles of allergic inflammation can be interrupted with effective early intervention, patients are much less likely to reach the latter stages where vision-threatening sequelae of cornea scarring and ectasia can occur [10,52].

## Figures and Tables

**Table 1:** VKC-specific TLR ligands and their expression at the ocular surface. * TLR2 forms heterodimers with TLR1 and TLR6 to initiate the signaling cascade [82].

TLR	Ligand	Pathogen
TLR2 (TLR1) *	Triacylated lipopeptides	Mycoplasma
TLR2 (TLR6) *	Diacylated lipopeptides	Gram-positive and Gram-negative bacteria
TLR4	LPS	Gram-negative bacteria
TLR8	ssRNA and imidazoquinolone antiviral drug	ssRNA viruses
TLR9	CpG-rich DNA	Gram-positive and Gram-negative bacteria; dsRNA viruses

## Data Availability

The original contributions presented in this study are included in the article. Further inquiries can be directed to the corresponding author.
